# Invasive Cancer Incidence and Survival — United States, 2013

**DOI:** 10.15585/mmwr.mm6603a1

**Published:** 2017-01-27

**Authors:** S. Jane Henley, Simple D. Singh, Jessica King, Reda J. Wilson, Mary Elizabeth O’Neil, A. Blythe Ryerson

**Affiliations:** 1 Division of Cancer Prevention and Control, National Center for Chronic Disease Prevention and Health Promotion, CDC.

Although cancer represents many heterogeneous diseases, some cancer types share common risk factors. For example, conclusive evidence links cancer at multiple sites with tobacco use, alcohol use, human papillomavirus (HPV) infection, excess body weight, and physical inactivity (*1*,*2*). To monitor changes in cancer incidence and assess progress toward achieving *Healthy People 2020* objectives,* CDC analyzed data from the U.S. Cancer Statistics (USCS) data set for 2013, the most recent year for which incidence and survival data are available. In 2013, a total of 1,559,130 invasive cancers were reported to cancer registries in the United States (excluding Nevada), for an annual age-adjusted incidence rate of 439 cases per 100,000 persons. Cancer incidence rates were higher among males (479) than females (413), highest among blacks (444), and ranged by state from 364 (New Mexico) to 512 (Kentucky) per 100,000 persons (359 in Puerto Rico). The proportion of persons with cancer who survived ≥5 years after diagnosis was 67%. This proportion was the same for males and females (67%), but lower among blacks (62%) than among whites (67%). Cancer surveillance data are key to cancer epidemiologic and clinical outcomes research, program planning and monitoring, resource allocation, and state and federal appropriations accountability.

The USCS data set is a compilation of data from multiple sources and is used to report official federal cancer statistics through the USCS web-based report. USCS includes high quality incidence data from population-based cancer registries affiliated with CDC’s National Program of Cancer Registries (NPCR) and the National Cancer Institute’s (NCI’s) Surveillance, Epidemiology, and End Results (SEER) program in each state, the District of Columbia (DC), and Puerto Rico; survival data from NPCR; and mortality data from the National Vital Statistics System (*3*,*4*). This report includes data on new cases of invasive cancer diagnosed in 2013 (the most recent year with data available); invasive cancers are all cancers excluding in situ cancers (except in the urinary bladder) and basal and squamous cell skin cancers. Data from DC and all states except Nevada met USCS publication criteria for 2013^†^; consequently, incidence data in this report cover 99% of the U.S. population. For comparability with past estimates, data for the United States were restricted to the states and DC, and data for Puerto Rico were analyzed separately. Cases were classified first by anatomic site, using the *International Classification of Diseases for Oncology, Third Edition*.^§^ Cases with hematopoietic histologies were classified further, using the *World Health Organization Classification of Tumours of Haematopoietic and Lymphoid Tissues, Fourth Edition*.^¶^ Breast cancers were characterized by stage at diagnosis using SEER Summary Staging Manual 2000**; late-stage cancers include those diagnosed after they had spread regionally or metastasized. To characterize the potential cancer prevalence associated with common risk factors, cancer sites were grouped by association with tobacco use, alcohol use, or HPV infection.^††^ Population denominators for incidence rates were annual race-, ethnicity-, and sex-specific county population estimates from the U.S. Census, as modified by NCI and aggregated to the state and national level.^§§^ Annual incidence rates per 100,000 population were age-adjusted to the 2000 U.S. standard population.

Survival estimates were based on data from NPCR-funded states that met USCS publication criteria and conducted active case follow-up or linkage with CDC’s National Center for Health Statistics National Death Index (*5*). For this report, 29 states met these criteria, covering 66% of the U.S. population. The 5-year relative survival proportion was defined as the proportion of persons surviving ≥5 years after cancer diagnosis compared with the proportion of survivors expected in a comparable group of cancer-free persons. The 5-year relative survival proportion was calculated using the Ederer II actuarial method for cases of cancer diagnosed during 2006–2012 with follow-up through 2012, accounting for shorter follow-up time of cases diagnosed in more recent diagnosis years.

In 2013, a total of 1,559,130 invasive cancers were diagnosed and reported to central cancer registries in the United States (excluding Nevada), including 781,451 among males and 777,679 among females (Table 1). The age-adjusted annual incidence for all cancers was 439 per 100,000 persons (479 in males and 413 in females). Among persons aged <20 years, 14,728 cancers (18 per 100,000 persons <20 years) were diagnosed in 2013 (Table 1). The cancer incidence rate increased with increasing age group, with highest rates (2,163 per 100,000 persons) among persons aged ≥75 years (Table 1).

**TABLE 1:** Number and annual age-adjusted rate* of invasive cancers,^†^ by sex, cancer site, race/ethnicity,^§^ and age group — National Program of Cancer Registries and Surveillance, Epidemiology, and End Results Program, United States,^¶^ 2013

Characteristic	Overall		Male		Female	
	Rate	No. (%)	Rate	No. (%)	Rate	No. (%)
**All cancer sites**	**439.0**	**1,559,130 (100)**	**479.0**	**781,451 (100)**	**412.6**	**777,679 (100)**
Prostate	**NA**	**176,450 (11)**	101.6	176,450 (23)	NA	NA
Female breast	**NA**	**230,815 (15)**	NA	NA	123.7	230,815 (30)
Late-stage female breast	**NA**	**76,816 (5)**	NA	NA	42.1	76,816 (10)
Lung and bronchus	**59.4**	**212,584 (14)**	69.8	111,907 (14)	51.5	100,677 (13)
Colon and rectum	**38.4**	**136,119 (9)**	44.2	71,099 (9)	33.6	65,020 (8)
Cervix uteri	**NA**	**11,955 (1)**	NA	NA	7.2	11,955 (2)
**Race/Ethnicity**						
White	**439.3**	**1,304,263 (84)**	473.9	654,240 (84)	417.4	650,023 (84)
Black	**443.6**	**170,123 (11)**	518.6	85,190 (11)	393.6	84,933 (11)
American Indian/Alaska Native	**276.4**	**8,676 (1)**	289.3	4,097 (1)	269.6	4,579 (1)
Asian and Pacific Islander	**284.0**	**47,802 (3)**	290.5	21,052 (3)	283.3	26,750 (3)
Hispanic	**343.7**	**117,332 (8)**	372.0	55,393 (7)	328.7	61,939 (8)
**Age group (yrs)**						
<20	**18.0**	**14,728 (1)**	18.7	7,815 (1)	17.3	6,913 (1)
20–49	**154.8**	**187,560 (12)**	111.9	68,160 (9)	197.2	119,400 (15)
50–64	**798.3**	**505,337 (32)**	837.0	258,164 (33)	763.2	247,173 (32)
65–74	**1,756.0**	**433,944 (28)**	2,075.9	239,204 (31)	1,477.0	194,740 (25)
≥75	**2,163.1**	**417,561 (27)**	2,685.9	208,108 (27)	1,813.7	209,453 (27)

**Abbreviation:** NA = not available.

*Per 100,000 persons, age-adjusted to the 2000 U.S. standard population.

^†^ Excludes basal and squamous cell carcinomas of the skin, except when these occur on the skin of the genital organs, and in situ cancers, except urinary bladder.

^§^ Racial categories are not mutually exclusive from Hispanic ethnicity. Rates are not presented for cases with unknown or other race. Hispanic category excludes cases from Virginia because a large percentage of cases were missing information on ethnicity.

^¶^ Compiled from data from cancer registries in 49 states and the District of Columbia that meet the data quality criteria for all invasive cancer sites combined (covering approximately 99% of the U.S. population).

By cancer site, incidence rates were highest for cancers of the female breast (124 per 100,000 females); prostate (102 per 100,000 males); lung and bronchus (lung) (59 per 100,000 persons); and colon and rectum (colorectal) (38 per 100,000 persons) (Table 1). These four sites accounted for approximately half of cancers diagnosed in 2013, including 230,815 female breast cancers, 176,450 prostate cancers, 212,584 lung cancers, and 136,119 colorectal cancers. In 2013, cervical cancer incidence was 7.2 per 100,000 females, representing 11,955 reported cancers (Table 1). The incidence (rates per 100,000 persons) for cancers associated with tobacco use, alcohol use, and HPV were 187, 130, and 11, respectively (Table 2).

**TABLE 2:** Annual age-adjusted rate* of invasive cancers,^†^ by cancer site and state — National Program of Cancer Registries and Surveillance, Epidemiology, and End Results program, United States, 2013

State	Cancer site						Cancers associated with certain risk factors^§^		
	All sites	Lung and bronchus	Colon and rectum	Prostate	Female breast	Cervix	Tobacco use	Alcohol use	HPV
Alabama	444.0	66.7	44.0	118.5	120.6	8.5	202.5	137.3	12.5
Alaska	410.4	54.7	43.0	76.6	120.5	6.6	187.2	130.0	11.6
Arizona	370.6	48.4	32.1	69.1	110.9	6.3	159.2	112.8	8.7
Arkansas	454.0	78.7	43.1	102.7	118.2	10.6	215.4	133.4	14.4
California	402.8	42.6	35.1	97.5	120.9	7.0	159.9	123.4	10.0
Colorado	396.1	42.2	33.6	101.6	123.6	5.7	152.3	120.5	9.2
Connecticut	474.2	62.3	36.3	104.6	138.4	7.3	196.1	136.4	11.6
Delaware	502.0	69.1	34.9	129.4	144.8	6.7	203.8	142.5	11.9
District of Columbia (DC)	445.2	55.3	41.4	120.1	138.2	8.3	181.4	144.0	13.0
Florida	413.0	58.8	35.8	86.4	114.1	8.4	179.0	123.4	13.4
Georgia	450.3	64.0	40.7	117.7	123.2	6.9	190.3	133.5	12.1
Hawaii	419.8	49.3	41.8	79.2	143.9	6.5	176.1	144.7	8.5
Idaho	419.5	46.9	35.1	101.4	119.4	5.2	169.1	120.8	10.5
Illinois	454.9	63.2	43.0	105.3	130.1	7.2	200.1	138.4	11.6
Indiana	438.8	71.6	42.1	85.7	120.4	7.6	205.2	132.0	12.4
Iowa	456.1	62.0	42.9	96.9	118.4	5.7	195.3	132.2	11.6
Kansas	450.9	61.8	38.6	108.5	115.6	7.1	189.6	125.3	10.8
Kentucky	511.7	93.4	49.0	104.8	123.2	7.9	244.5	146.1	14.1
Louisiana	476.3	68.0	45.0	131.2	124.6	8.2	215.0	142.6	12.7
Maine	463.8	74.8	37.4	80.7	126.0	5.9	203.0	130.1	12.4
Maryland	451.0	56.6	35.8	124.4	134.1	5.9	180.3	133.6	9.8
Massachusetts	457.5	62.6	36.4	97.2	137.2	4.9	192.7	137.7	10.8
Michigan	440.1	62.4	36.7	102.8	124.8	6.7	189.0	128.0	10.8
Minnesota	451.8	56.6	38.7	101.6	127.9	5.3	180.6	129.9	9.4
Mississippi	459.9	75.2	48.7	127.2	112.3	8.1	218.7	139.2	13.0
Missouri	442.6	73.7	41.0	84.7	124.9	7.7	204.9	134.8	12.4
Montana	437.0	58.0	38.8	108.0	109.4	4.1	180.0	119.1	9.0
Nebraska	437.6	60.1	39.9	106.1	118.7	7.4	184.3	125.1	10.1
Nevada	—^¶^	—^¶^	—^¶^	—^¶^	—^¶^	—^¶^	—^¶^	—^¶^	—^¶^
New Hampshire	479.2	64.8	37.0	115.6	148.4	3.6	192.0	140.3	10.2
New Jersey	483.1	57.5	41.9	123.4	135.5	7.5	193.1	139.6	10.9
New Mexico	363.7	39.6	32.1	80.9	112.6	7.2	148.3	115.7	9.0
New York	484.3	59.5	39.3	125.6	130.3	8.0	196.2	135.7	11.2
North Carolina	445.4	68.5	36.3	107.8	126.1	6.6	194.5	131.4	11.7
North Dakota	433.6	56.4	46.0	100.5	125.5	—**	183.8	132.3	8.8
Ohio	452.4	67.4	40.6	101.7	125.8	7.4	200.3	134.3	12.1
Oklahoma	440.3	68.7	42.5	95.0	117.0	9.5	203.0	132.7	13.0
Oregon	431.5	55.6	34.5	88.7	124.8	6.7	179.9	126.8	12.1
Pennsylvania	483.0	64.3	42.3	101.2	130.8	7.1	204.6	139.8	11.8
Rhode Island	479.4	69.9	35.4	91.1	137.8	7.4	206.5	136.3	12.1
South Carolina	436.9	64.4	36.1	105.8	124.5	7.7	191.3	130.7	13.2
South Dakota	450.1	59.4	40.8	102.1	146.1	7.3	183.3	139.1	11.5
Tennessee	450.9	74.1	38.2	106.2	124.6	8.9	203.0	133.4	13.3
Texas	399.4	52.7	37.4	88.6	108.4	8.7	177.5	122.4	10.5
Utah	393.2	26.1	32.0	111.9	111.0	4.8	128.1	107.5	7.2
Vermont	437.1	59.1	31.7	81.7	125.6	5.7	176.7	123.5	10.4
Virginia	418.5	58.2	35.4	101.0	128.3	5.8	175.5	128.3	10.0
Washington	450.3	55.0	35.3	107.3	135.3	6.7	179.9	132.8	10.8
West Virginia	464.0	79.1	47.0	90.3	116.5	8.1	223.0	137.1	12.9
Wisconsin	451.1	59.0	36.4	103.4	128.6	5.5	187.5	129.5	9.7
Wyoming	382.0	38.7	32.5	97.9	105.0	6.0	145.9	106.7	8.3
Puerto Rico (PR)	358.5	18.2	42.0	144.9	95.8	12.3	124.3	117.4	12.3
States + DC + PR	439.0	58.8	38.5	102.1	123.4	7.3	186.2	130.2	11.3
States + DC	438.0	59.4	38.4	101.6	123.7	7.2	187.0	130.4	11.2

**Abbreviation:** HPV = human papillomavirus.

* Age-adjusted to the 2000 U.S. standard population. Rates are per 100,000 persons except per 100,000 males for prostate cancer and per 100,000 females for female breast and cervical cancers.

^†^ Excludes basal and squamous cell carcinomas of the skin, except when these occur on the skin of the genital organs, and in situ cancers, except urinary bladder.

^§^ Tobacco-associated cancers include oral cavity and pharynx; esophagus; stomach; colon and rectum; liver; pancreas; larynx; lung, bronchus, and trachea; cervix; kidney and renal pelvis; urinary bladder; and acute myeloid leukemia. Alcohol-associated cancers include oral cavity and pharynx; esophagus; colon and rectum; liver; larynx; and female breast. HPV-associated cancers include microscopically confirmed carcinoma of the cervix and squamous cell carcinomas of the vagina, vulva, penis, anus, rectum, and oropharynx.

^¶^ Rate not shown because data did not meet publication criteria.

** Rate not shown because <16 cases were reported.

By state, in 2013, age-adjusted incidence rates for cancers of all sites (all-sites cancer) ranged from 364 per 100,000 persons in New Mexico to 512 per 100,000 persons in Kentucky (Table 2). State site-specific cancer incidence rates for prostate cancer ranged from 69 (Arizona) to 131 (Louisiana) per 100,000 males; for female breast cancer, from 105 (Wyoming) to 148 (New Hampshire) per 100,000 females; for lung cancer, from 26 (Utah) to 93 (Kentucky) per 100,000 persons; for colorectal cancer, from 32 (Arizona, New Mexico, Utah, and Vermont) to 49 (Kentucky and Mississippi) per 100,000 persons; and for cervical cancer, from four (New Hampshire and Montana) to 11 (Arkansas) per 100,000 females (Table 2). The *Healthy People 2020* target for reducing colorectal cancer incidence to ≤39.9 per 100,000 persons was reached in 30 states and the target for reducing cervical cancer incidence to ≤7.2 per 100,000 females was reached in 28 states. For grouped cancers, incidence rates for tobacco-related cancers ranged by state from 128 (Utah) to 245 (Kentucky) per 100,000 persons; for alcohol-related cancers, from 107 (Wyoming) to 146 (Kentucky) per 100,000 persons; and for HPV-related cancers, from seven (Utah) to 14 (Arkansas and Kentucky) per 100,000 persons (Table 2). Compared with the states and DC, cancer incidence rates in Puerto Rico in 2013 were lower for all-sites cancer (359 per 100,000 persons), lung cancer (18 per 100,000 persons), and female breast cancer (96 per 100,000 females), but higher for prostate cancer (145 per 100,000 males), colorectal cancer (42 per 100,000 persons), and cervical cancer (12 per 100,000 females) (Table 2).

Among persons with cancer diagnosed during 2006–2012, the 5-year relative survival proportion was 67% (Table 3). This proportion was similar for males and females. The 5-year relative survival proportion was highest among persons who received a diagnosis of cancer before age 45 years (83%) and decreased with increasing age (Table 3). Among the four most common cancer sites, the 5-year relative survival proportion was highest for prostate cancer (99%) and female breast cancer (90%), intermediate for colorectal cancer (66%), and lowest for lung cancer (19%) (Table 3). The 5-year relative survival proportion after any cancer diagnosis was lower among blacks (62%) than among whites (67%), particularly among black females (59%) compared with white females (68%) (Table 3).

**TABLE 3:** Percentage of patients with five-year relative survival after cancer diagnosis,* by race, sex, cancer site, and age group — National Program of Cancer Registries, 29 States, 2006–2012^†^

Cancer site/Age group	Survival (%)								
	All races			Whites			Blacks		
	Overall	Males	Females	Overall	Males	Females	Overall	Males	Females
**All sites**	**67**	**67**	**67**	**67**	**67**	**68**	**62**	**64**	**59**
Prostate	NA	99	NA	NA	99	NA	NA	97	NA
Female breast	NA	NA	90	NA	NA	91	NA	NA	80
Lung and bronchus	19	17	23	19	17	23	17	14	20
Colon and rectum	66	65	66	66	66	67	59	58	61
Cervix uteri	NA	NA	69	NA	NA	70	NA	NA	59
**Age group (yrs)^§^**									
<45	83	78	85	84	80	87	72	67	75
45–54	74	69	78	75	70	80	65	63	66
55–64	71	70	71	71	70	72	64	67	61
65–74	66	69	63	67	69	64	62	67	54
≥75	53	56	50	53	55	51	46	52	42

**Abbreviation:** NA = not applicable.

* Based on cases of cancer diagnosed during 2006–2012 and follow-up of patients through 2012.

^†^ Compiled from data from 29 cancer registries that met data quality criteria for survival analysis, covering approximately 66% of the U.S. population.

^§^ Age at cancer diagnosis.

## Discussion

This report provides estimates of cancer incidence for 2013 in the United States and indicates that national *Healthy People 2020* targets were achieved in 30 states for reduced colorectal cancer incidence and 28 states for reduced cervical cancer incidence. Many cancers could be prevented by implementing evidence-based interventions to reduce cancer risk factors, promote healthy living, and encourage appropriate cancer screening (*6*).

Some cancer risk factors can be addressed through clinical preventive services. As of 2016, the U.S. Preventive Services Task Force recommends that all adults be screened for tobacco use and excessive alcohol use and offered counseling and intervention as needed.^¶¶^ The U.S. Preventive Services Task Force also recommends the use of low-dose aspirin to prevent colorectal cancer and cardiovascular disease among adults who are considered to be at high risk for cardiovascular disease based on specific criteria (*7*). As of 2016, the Advisory Committee on Immunization Practices recommends vaccination against two cancer-causing viruses, HPV and hepatitis B virus.*** Health care providers play an important role in ensuring that all children, adolescents, and adults receive the preventive services they need at the appropriate time. The Community Preventive Services Task Force offers evidence-based recommendations to increase both patient and provider adherence to guidelines for preventive services as well as community-based approaches to promote physical activity, reduce excessive alcohol use, and reduce tobacco use and tobacco smoke exposure.^†††^

Cancer incidence and survival data are important for guiding the planning and evaluation of cancer prevention and control programs at the national and local levels. For example, Pennsylvania Cancer Registry data were used to guide community outreach programs in areas with cancer-related health disparities and evaluate the impact of cancer interventions (*8*). These data also assist long-term planning for cancer diagnostic and treatment services. By examining prostate cancer treatment data, the New Hampshire State Cancer Registry found promising trends in the management of prostate cancer, with increasing use of surgical procedures for men at high risk for the disease and less aggressive treatment for men at low risk for the disease (*9*). Finally, these data help public health officials set priorities for allocating health resources. The Oregon Health Authority’s decision to increase measures to improve HPV vaccination coverage was based in part on data from the Oregon State Cancer Registry that indicated a recent increase in HPV-associated cancer incidence had occurred (*10*).

CDC annually provides cancer surveillance data via several products such as USCS, CDC WONDER, State Cancer Facts, and the CDC Chronic Disease Indicators web tool, and through the CDC’s National Center for Health Statistics Research Data Center.^§§§^ Although cancer mortality data sets formatted for use with NCI SEER*Stat software have been available since 2003, for the first time, CDC is releasing a public use NPCR cancer incidence data set that can be analyzed using the NCI SEER*Stat software; information about this data set, including variable formats for cancer groups related to tobacco use, alcohol use, HPV, obesity, and physical activity, is available online.^¶¶¶^

The findings in this report are subject to at least four limitations. First, analyses based on race/ethnicity might be biased if race/ethnicity was systematically misclassified; ongoing procedures are used to ensure that this information is as accurate as possible (*4*). Second, delays in cancer reporting might result in an underestimation of certain cancers; reporting delays are more common for cancers such as melanoma and prostate cancer that are diagnosed and treated in nonhospital settings such as physicians’ offices. Third, relative survival could only be calculated for white and black racial groups because accurate life tables were not available for other racial/ethnic groups. Finally, because information about risk factors is not routinely collected by cancer registries, estimates for risk factor–associated cancers depict the number potentially associated, not the number definitively attributable.

Public health officials use population-based cancer incidence, mortality, and survival surveillance data to plan and monitor programs, conduct clinical outcomes research, help make decisions about allocating resources, and hold recipients of state and federal appropriations accountable. To achieve the national cancer objectives set forth in *Healthy People 2020*, initiatives to promote healthy living, reduce exposure to cancer risk factors, improve adherence to cancer screening recommendations, and assure timely and appropriate clinical preventive services for all persons should be maximized. The effects of these initiatives can be monitored using cancer surveillance data.

**Summary**

**What is already known about this topic?**

Some risk factors, such as tobacco use, alcohol use, excess body weight, physical inactivity, and human papillomavirus (HPV) infection increase the risk for more than one type of cancer.

**What is added by this report?**

Based on the U.S. Cancer Statistics dataset, in 2013 (the most recent year for which incidence and survival data are available), a total of 1,559,130 new invasive cancers were diagnosed in the United States (excluding Nevada), for an annual incidence of 479 per 100,000 males and 413 per 100,000 females. All-sites cancer incidence rates ranged, by state, from 364 to 512 per 100,000 persons, and the rate was 359 per 100,000 persons in Puerto Rico. *Healthy People 2020* targets for reducing incidence rates were reached in 30 states for colorectal cancer and 28 states for cervical cancer. Approximately two of three persons survived ≥5 years after cancer diagnosis.

**What are the implications for public health practice?**

Cancer surveillance data are key to cancer epidemiologic and clinical outcomes research, program planning and monitoring, resource allocation, and state and federal appropriations accountability. To achieve the national cancer objectives set forth in *Healthy People 2020,* initiatives to promote healthy living, reduce exposure to cancer risk factors, improve adherence to cancer screening recommendations, and assure timely and appropriate clinical preventive services and treatment for all persons must be maximized. The impact of these initiatives can be monitored using publicly available cancer surveillance data.

## Footnotes

* As of 2017, *Healthy People 2020* objectives included improving the proportion of persons surviving ≥5 years after cancer diagnosis to 71.7%, reducing colorectal cancer incidence to 39.9 per 100,000 persons, reducing late-stage breast cancer incidence to 42.1 per 100,000 females, and reducing cervical cancer incidence to 7.2 per 100,000 females (https://www.healthypeople.gov/2020/topics-objectives).

† Cancer registries demonstrated that cancer incidence data were of high quality by meeting the six USCS publication criteria: 1) case ascertainment ≥90% complete; 2) ≤5% of cases ascertained solely on the basis of death certificate; 3) ≤3% of cases missing information on sex; 4) ≤3% of cases missing information on age; 5) ≤5% of cases missing information on race; and 6) ≥97% of registry’s records passed a set of single-field and interfield computerized edits that test the validity and logic of data components (https://www.cdc.gov/cancer/npcr/uscs/index.htm).

§ http://codes.iarc.fr/.

¶ https://www.ncbi.nlm.nih.gov/pmc/articles/PMC3109529/.

** https://seer.cancer.gov/tools/ssm.

†† Tobacco-associated cancers include cancers of the oral cavity and pharynx; esophagus; stomach; colon and rectum; liver; pancreas; larynx; lung, bronchus, and trachea; cervix; kidney and renal pelvis; urinary bladder; and acute myeloid leukemia. Alcohol-associated cancers include cancers of the oral cavity and pharynx; esophagus; colon and rectum; liver; larynx; and female breast. HPV-associated cancers include microscopically confirmed carcinoma of the cervix and squamous cell carcinomas of the vagina, vulva, penis, anus, rectum, and oropharynx.

§§ Population estimates incorporate bridged single-race estimates derived from the original multiple race categories in the 2010 U.S. Census. https://seer.cancer.gov/popdata/index.html; http://www.census.gov/programs-surveys/popest.html.

¶¶ https://www.uspreventiveservicestaskforce.org/Page/Name/recommendations.

*** https://www.cdc.gov/vaccines/hcp/acip-recs/vacc-specific/hpv.html; https://www.cdc.gov/vaccines/hcp/acip-recs/vacc-specific/hepb.html.

†††
https://www.thecommunityguide.org/topic/cancer .

§§§ https://www.cdc.gov/cancer/npcr/datarelease.htm; https://wonder.cdc.gov; https://www.statecancerprofiles.cancer.gov/incidencerates/; https://www.cdc.gov/rdc/b1datatype/dt131.htm; https://www.cdc.gov/cdi.

¶¶¶ https://www.cdc.gov/cancer/npcr/public-use.
